# Thoracic index in adults with asthma: a study of validity and reliability

**DOI:** 10.1186/s12998-018-0187-x

**Published:** 2018-05-24

**Authors:** Yannely Serrano-villar, Eliana-isabel Rodríguez-grande

**Affiliations:** 1 Universidad Industrial de Santander Facultad de Salud, Bucaramanga, Santander Colombia; 2 Sede Quinta Mutis, Escuela de Medicina y Ciencias de la Salud, Universidad del Rosario, Carrera 24N # 63D-69, Bogotá, Colombia

**Keywords:** Asthma, Thoracic index, Reproducibility, Validity, Costal mobility, Respiratory mechanics

## Abstract

**Background:**

The Thoracic Index (TI) is a useful tool for evaluating costal mobility as a component of respiritory mechanics in adults with asthma. In a review of the literature, however, few studies were found that reported the psychometrics of this test. The goal of this study is to evaluate the reproducibility and validity of the TI in adults with asthma.

**Methods:**

A cross-sectional study was conducted to evaluate the diagnostic tests. Measurements were done randomly by two independent evaluators. The variables measured included thoracic mobility (TI and photogrammetric analysis), sociodemographic and anthropometric variables, and other variables related to the disease. TI reliability included the determination of the intra- and inter-evaluator agreement and reproducibility using the Bland and Altman limits of agreement method and the Interclass Correlation Coefficient (ICC). The convergent validity was established using Pearson’s correlation coefficient. The level of significance was *p* < 0.05.

**Results:**

Twenty-six adults with stable asthma participated in this study. The limits of the intra- and inter-evaluator agreement were found to be acceptable and good, respectively, with an average of differences close to zero in both cases. The intra-evaluator reproducibility was between poor and acceptable (TI between 0.57 and 0.93), while the inter-evaluator reproducibility was between acceptable and good (TI between 0.62 and 0.86). The convergent validity between the TI and photogrammetric analysis was between moderate and high (r between 0.55 and 0.73).

**Conclusions:**

The TI is a reliable and valid measurement that can be used to evaluate costal mobility in adults with asthma. In a clinical setting, it can contribute to a nonbiased measurement, and in a research environment, it is useful for documenting the results of interventions, reducing the probability that the results will be affected by any variability in measurement.

## Background

Asthma is considered the world’s most common chronic respiratory disease. It affects 334 million people of all ages and is the 14th most important disorder in the world in terms of its extent and duration of disability [1]. It is characterized by a chronic inflammation of the airways, limiting the expiratory airflow, which produces intermittent hyperinflation and adaptations in the thoracic cage to move the trapped air, with reduced costal mobility [2–4].

The evaluation of thoracic mobility allows us to quantify the functional consequences and the degree to which asthma is controlled [3]. Photogrammetry is one of the tools available for this evaluation, providing a kinematic analysis of respiratory movements, but it requires special equipment and training, and the computerized analysis of images requires additional time per patient, increasing its cost and minimizing its clinical applicability [5–7]. Thoracic perimetry is used in a clinical context, expressed as the Thoracic Index (TI), because it is a low-cost and easy-to-apply technique. In a review of the literature, however, no studies were found that evaluated the TI’s psychometric properties in adults with stable asthma [8–11].

Having reliable and valid measuring tools in a clinical setting allows the clinician to provide an objective evaluation and diagnosis, and to undertake appropriate interventions to improve the respiratory mechanics compromised by asthma [2]. They are also useful in a research context for demonstrating the results of physiotherapeutic interventions, decreasing the likelihood that the effects obtained will be influenced by any variability in the measurements [9].

For these reasons, it is necessary to evaluate the psychometric properties of the tests used in clinical practice, using tests and measurements of proven validity and reproducibility as test comparators. The reliability and validity of the TI are tested against a photogrammetric analysis of the breathing cycle. This will allow an improved evaluation of the respiratory mechanics in asthma. It will also allow the control of the disease to be monitored and the provision of an evidentiary basis for intervention programs to control symptoms, prevent complications, and improve the functionality and quality of life of this population [12, 13].

The objective of this study was to evaluate the intra- and inter-evaluator reliability of TI measurements, and the convergent validity between the TI and photogrammetric analysis in a population of asthmatic adults.

## Method

A study was conducted to evaluate the reliability and validity of diagnostic tests using a cross-sectional sample [14].

### Subjects

The subject group included adults with stable asthma. People with comorbidities such as the following were excluded: cardiac disease, uncontrolled arterial hypertension, post-operative from lung biopsy, spinal injury, ocular injury, tracheotomy, upper respiratory tract surgery or trauma, hemodynamic instability, pregnancy, or respiratory infections [15] with musculoskeletal or neurological sequels or diseases that compromised thoracic mobility and muscular control [16], with a lack of voluntary force during spirometry, defined as the peak expiratory flow (PEF) or forced expiratory flow (FEF) at 25%, below the 60% normal value [17–19]. The measurements were taken at the Movement Analysis Laboratory at the School of Physiotherapy at the Universidad Industrial de Santander.

### Evaluators

Two physical therapists with clinical experience of between 5 and 13 years participated in this study. They standardized their verbal instructions and manual contact with the subjects and were trained in applying the tests to avoid classification bias.

### Procedure

**Fig. 1:**
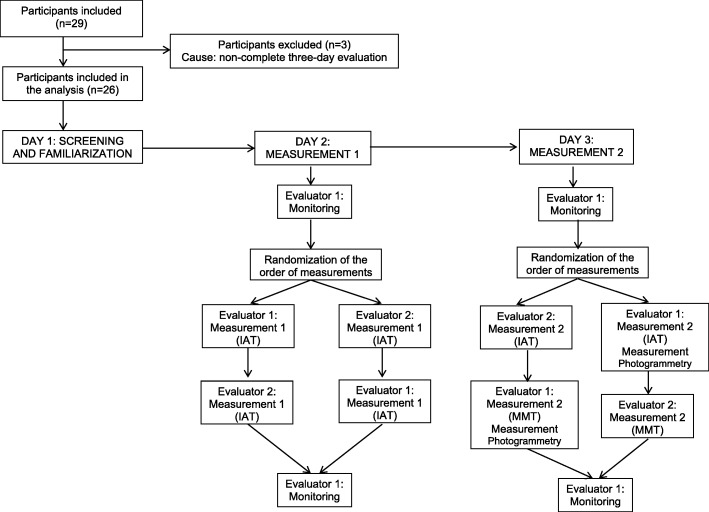
Flowchart of study participants

The protocol consisted of measurements that were taken on three days (between 2 and 8 days apart). The screening and familiarization with the object of the study were carried out on the first day, including the anthropometric and spirometric measurements, the evaluation of the sociodemographic variables and the variables related to the disease and its monitoring, as well as familiarization with the variables of thoracic mobility. The TI was measured independently by the two evaluators on the second day. The measurement of the TI by the evaluators was repeated on the third day. An evaluator also conducted the photogrammetry on the third day. The thoracic mobility variables were measured in random order, and an evaluator monitored the participants at the beginning and end of each session. The two evaluators were blinded to previous measurements and to the measurements of the other evaluator, and the evaluations were carried out at the same time of day. The subjects were asked to continue their medical treatment for the duration of the study (Fig. 1).

### Measurements

#### Spirometry

A Spirobank G brand MIR SRL spirometer was used; the technical procedures recommended by the American Thoracic Society and the European Respiratory Society were followed [17, 20]. The subjects exhaled as hard as they could at least three times. Their peak expiratory flow (PEF) was measured, as was their forced expiratory flow, (FEF)_25%_. If the PEF was under 60% of the predicted value [17, 18, 20], the participant was excluded from the study. The volume exhaled at the end of the first second (FEV_1_) was also noted, as was the forced vital capacity (FVC) and the ratio FEV_1_/FVC.

#### Anthropometric variables

Body weight was measured using a portable digital scale. Height was measured to an accuracy of 1 mm using a non-stretchable metal tape measure. Recommendations for the anthropometric procedures were followed as stated in the National Health and Nutrition Examination Survey manual published by the Centers for Disease Control and Prevention [21]. Height was measured in meters (m) and weight in kilograms (Kg), and their values were used to calculate the body mass index (BMI, weight/height^2^) [22, 23].

#### Monitoring variables

Vital signs were measured (heart rate in beats per minute; respiratory rate in breaths per minute; and arterial tension in millimeters of mercury) [24, 25]. Oxygen saturation was measured to an accuracy of 2% [26]. The findings from pulmonary auscultation and observed respiratory difficulty as measured by the Borg Scale [25, 27] were also recorded.

#### Variables of thoracic mobility

Thoracic mobility was measured using the TI and photogrammetric analysis. The evaluation was conducted with the participants in a sitting position in a backless chair with their feet flat on the floor and arms resting on adjustable-height armrests. The thorax was uncovered for the men and partially uncovered for the women. Thoracic circumference was measured using a non-stretchable metal tape measure at three levels in the rib cage. Reflective markers were placed on the anatomical structures at each level, including the axillary level, the third anterior intercostal space in the front, and the fifth thoracic spinous process in the back; at the level of the xiphoid, the xiphoid process in the front and the tenth thoracic spinous process in the back; at the umbilical level, the umbilical scar in the front and the third thoracic spinous process in the back. The procedure was standardized, putting zero on the metal tape measure at the corporal midpoint at the levels of the respective reflective markers. The participants were requested to take a maximally deep breath and then to exhale maximally. The tape measure was adjusted at the end of the inhalation and again at the end of the exhalation, and the result in centimeters was obtained by finding the difference between the diameter at the maximal inhalation and the diameter at the maximal exhalation [9, 11, 28].

Measurements were taken twice at each level; the higher of the two measurements and the average of the two measurements were recorded.

For the photogrammetric analysis, reflective markers were placed at the same levels as for the TI measurement, and photographic images were taken of the participants at maximum inhalation and maximum exhalation. As with the measurements, two photographs were taken, and each one was processed using Software for Postural Evaluation (SAPO). Thoracic-abdominal displacement in centimeters was obtained at the axillary, xiphoidal, and abdominal levels for the maximum and average values of each measurement [29].

### Statistical analysis

Having taken two measurements at each level, assuming an explanatory power of 80% and a significance level of 5% with a loss percentage of 20% [30], it was determined that between 25 and 35 people would be sufficient to assess the psychometric properties.

The central tendency and dispersion measures were applied to characterize the population based on the nature and distribution of the variables. The analysis of the TI’s reliability included determining the level of agreement and reproducibility, both intra- and inter-evaluator, using the Bland and Altman limits of agreement method [31] and the Interclass Correlation Coefficient (ICC 2, K), thus establishing their respective Confidence Intervals at 95% (CI95%). The TI was categorized as follows: good reproducibility between 0.8 and 1.0; fair reproducibility between 0.6 and 0.79; poor reproducibility ICC < 0.6; and clinically acceptable > 0.7 [9]. The convergent external validity was established using Pearson’s correlation coefficient. The expected level of correlation was classified in keeping with the results of Carter et al. as follows: little (*r* = 0.0–0.25), low (*r* = 0.26–0.49), moderate (*r* = 0.50–0.69), high (*r* = 0.70–0.89), and very high (*r* = 0.90–1.00).

## Results

### Participant flow

Twenty-nine adults bwith stable asthma were included in the study; three of them did not complete the required evaluations because they did not attend the three measurement days, so 26 were included in the definitive analysis (Fig. 1); 16/26 (61.54%) were women. Table 1 describes the sample in terms of the general spirometric characteristics and characteristics related to the disease. The spirometric pattern was determined to be normal for all the participants based on FEV_1_ and FVC exceeding 80% of the predicted value, and the FEV_1_/FVC relation over 70% of the predicted value [20].

**Table 1:** Characteristics of the sample (*N* = 26)

Variable	Data
Age, years (percentile 25;75)	24.5 (20;32)
Weight, kg (SD)^a^	69.4(16.11)
Size, m (SD)	1.6 (0.08)
BMI^b^, Kg/m^2^ (SD)	25.5 (5.24)
Schooling, approved years (SD)	14.6 (3.43)
Asthma Features	
Time of disease evolution, years (SD)	18.1 (9.31)
Time after last acute exacerbation, months (percentile 25;75)	7 (4;36)
Spirometric values	
PEF^c^ (SD)	93.3 (20.88)
FEF_25%_^d^ (SD)	74.1 (23.66)
FEV_1_^e^ (SD)	82.8 (13.82)
FVC^f^ (SD)	91.2 (9.79)
FEV_1_/FVC (SD)	89.8 (10.92)

^a^Standard Deviation

^b^Body Mass Index

^c^Peak Expiratory Flow

^d^Forced Expiratory Flow at 25% of FVC

^e^Forced Expiratory Volume during the first second

^f^Forced Vital Capacity

### TI reliability

**Fig. 2:**
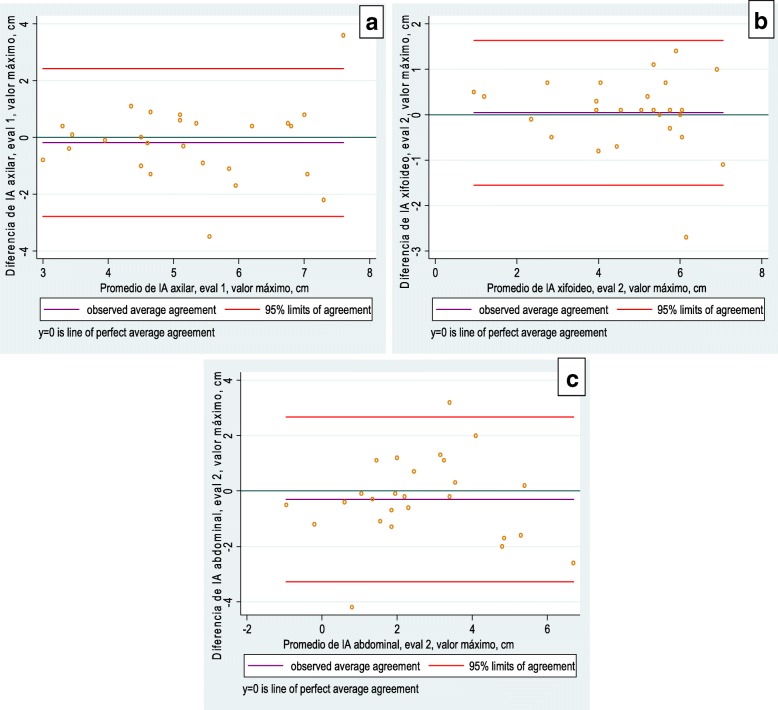
Levels of intra-evaluator agreement. **a** Evaluator 1, axillary level. **b** Evaluator 2, xiphoid level. **c** Evaluator 2, abdominal level

The analysis of the intra-evaluator agreement showed acceptable limits of agreement for two evaluators, with the average of the differences between them close to zero, using both the maximum value and the average (Fig. 2). In the intra-observer analysis, the Bland Altman plots showed a limits of agreement narrowest at the xiphoid level (Evaluator 1: 1.892 and 1.576; Evaluator 2: 1.006 and 1.206) and greater difference at the abdominal level (Evaluator 1: 3.519 and 2.888; Evaluator 2: 3.038 and 2.415).

**Fig. 3:**
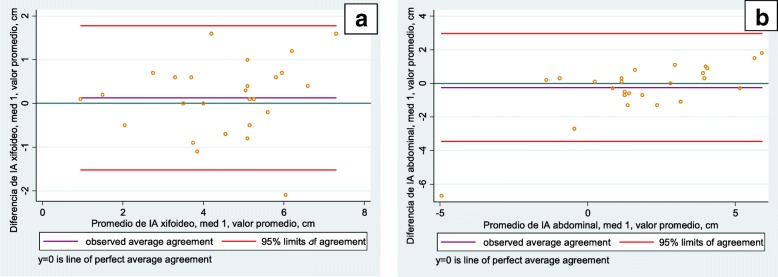
Level of agreement between evaluators, average value. **a** Xiphoid level. **b** Abdominal level

**Fig. 4:**
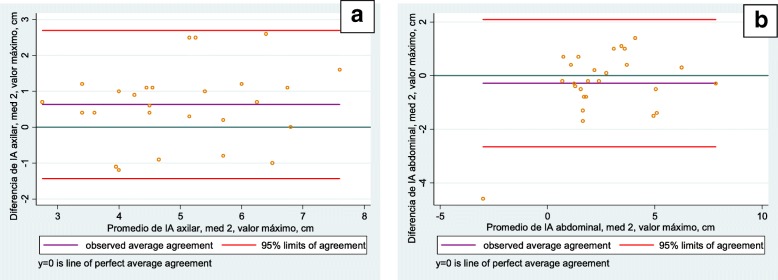
Level of agreement between evaluators, maximum value. **a** Axillary level. **b** Abdominal level

In the inter-observer analysis the Bland-Altman plots showed a smaller difference at the xiphoid level (measurement 1: 1.787 and 1.917; measurement 2: 1.773 and 2.342) and greater at the abdominal level (measurement 1: 3.638 and 3.100; measurement 2: 2.659 and 2.090). Only 3.23% of the data were outside the limits of agreement, representing one participant at the xiphoid level and one at the abdominal level, both on the first measurement day. The following figures illustrate a similar data distribution, without significant bias (Figs. 3 and 4). In general, there was good intra-and inter-evaluator agreement at the three levels of measurement [9].

### TI reproducibility

The intra-evaluator reproducibility for measurement at the axillary level was between poor and acceptable (ICC between 0.57 and 0.68), analyzing the maximum and average values, respectively. At the xiphoid level it was good (ICC between 0.85 and 0.93) and at the abdominal level it was between acceptable and good (ICC between 0.70 and 0.82), with wider confidence intervals at the axillary and abdominal levels. The reproducibility between the evaluators was acceptable at the axillary level both for the maximum and average values (ICC between 0.62 and 0.66), and good at the xiphoid and abdominal levels (ICC between 0.78 and 0.86), with wider confidence intervals at the axillary level (Tables 2 and 3).

**Table 2:** Intra-evaluator TI reproducibility (*N* = 26)

Measurement level	Evaluator 1		Evaluator 2	
	ICC [2,k]	IC 95%	ICC [2,k]	IC 95%
Maximum value				
Axillary	0.596	0.279–0.796	0.571	0.237–0.783
Xiphoid	0.856	0.708–0.933	0.885	0.760–0.947
Abdominal	0.784	0.577–0.897	0.704	0.446–0.855
Average value				
Axillary	0.688	0.422–0.846	0.631	0.324–0.817
Xiphoid	0.858	0.711–0.933	0.935	0.863–0.970
Abdominal	0.820	0.641–0.914	0.718	0.469–0.862

The data are the reproducibility for each evaluator. ICC: Interclass Correlation Coefficient; IC: Confidence Interval

**Table 3:** Inter-evaluator TI reproducibility (N = 26)

Measurement level	Inter-evaluator TI reproducibility in Measurement 1		Inter-evaluator TI reproducibility in Measurement 2	
	ICC [2,k]	IC 95%	ICC [2,k]	IC 95%
Maximum value				
Axillary	0.644	0.324–0.827	0.629	0.261–0.825
Xiphoid	0.845	0.684–0.927	0.806	0.618–0.908
Abdominal	0.787	0.583–0.898	0.850	0.696–0.929
Average value				
Axillary	0.664	0.376–0.835	0.634	0.243–0.832
Xiphoid	0.865	0.724–0.937	0.853	0.668–0.935
Abdominal	0.793	0.592–0.901	0.841	0.680–0.925

The data are the reproducibility between the evaluators on each measurement day

*ICC* Interclass Correlation Coefficient, *IC* Confidence Interval

### Convergent validity between the TI and thoracic kinematics

**Fig. 5:**
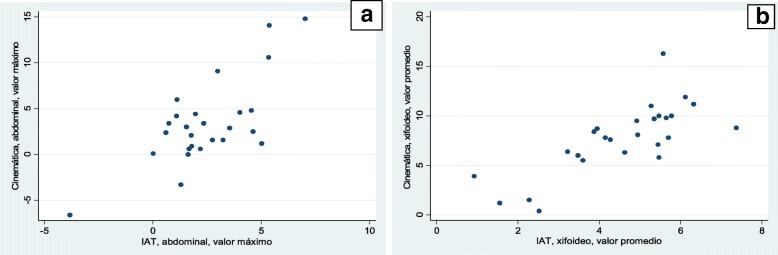
Relation between kinematics and TI. **a** Abdominal level, maximum value. **b** Xiphoid level, average value

The correlation using the maximum value was moderate at the axillary and xiphoid levels, and high at the abdominal level (r axillary: 0.621; xiphoid: 0.668; abdominal: 0.733), with narrower confidence intervals at the abdominal level. The correlation using the average value was moderate at the axillary level (r: 0.55) and high at the xiphoid and abdominal levels (r: 0.757 and 0.696, respectively), with narrower confidence intervals at the xiphoid level (Fig. 5).

## Discussion

Within the established reliability, the limits to agreement were narrower at all the measurement levels than those reported by Malaguti et al. [9], who evaluated the reproducibility of thoracic cirtometry in people with COPD. This result can be attributed to the standardization of all the conditions within the protocol. The limits were considered acceptable both for the intra-evaluator and inter-evaluator agreements, using the maximum and average values with an average of differences close to zero, thus demonstrating control of measurement bias, possibly resulting from the session where the evaluators were trained and the participants were familiarized with the measurements.

In both studies, the limits were narrowest at the xiphoid level and widest apart at the abdominal level. Malaguti et al. [9] attributed the greater variability at the abdominal level to differences in the breathing patterns of each participant at maximum inhalation. In addition, when analyzing the variability at the abdominal level in the seated position, the distribution of the pulmonary volumes should be taken into consideration. Lee et al. [32] concluded that subtle changes in the trunk position of healthy adults can alter the configuration and movement of the thoracic wall, as well as the distribution of the respiratory volume, changes that can be attributed to modifications in muscular activation [33].

These modifications in the neck and trunk muscles are related to the double function of these muscles in the respiratory cycle and postural control [34, 35], especially at the abdominal level. Postural adjustments in a seated position can influence breathing patterns due to the alternation of these functions. Small postural changes can favor the predominance of either the postural or the respiratory function [33, 36]. Thus, despite having a standardized seated posture for every participant, small postural adjustments may have influenced the variability of the measurements.

Romei et al. [6] found that costal kinematics in healthy adults are significantly affected by the trunk position. A gradually increasing inclination of the trunk leads to a progressive reduction in rib cage displacement and an increasing abdominal contribution leads to tidal volume. These findings may also explain the variable TI measurements for individual subjects, and indicate aspects of intra-subject measurements that can generate random errors outside the evaluator’s control.

In addition, the measurement of costal mobility has been used to determine the effectiveness of physiotherapeutic interventions for people with asthma. Burianová et al. [37] evaluated the effect of physiotherapeutic treatment on thoracic mobility and reported a statistically significant improvement with total increments of between 1.5 cm and 2.1 cm for men at the level of the fourth rib and xiphoid level, respectively, and of 2 cm for women at both levels. The findings of this study, however, suggest that changes under 2.1 cm cannot be considered clinically significant, as they can represent randomization given the variability of measurements for either the evaluated subject or the evaluator rather than the effect of the intervention, which is highly relevant in the clinical practice of physiotherapy.

With regard to reproducibility, a wide IC of 95% was found at every level, which should be analyzed in light of the participants’ pathological condition. In asthma, hyperinflation takes place during the crisis. When the disease recedes, breathing patterns may vary from person to person [38]. Thus, greater variations in costal mobility may appear in asthma patients than in people with COPD, which could explain the lesser reproducibility at the axillary level in this study compared to Malaguti et al. [9].

The intra-evaluator reproducibility of between acceptable and good may be attributable to the influence of sex over mobility. Romei et al. [6] showed that the abdominal contribution to tidal volume is less among women, so including subjects of both sexes in this study may have introduced an additional source of variability. The participants’ posture during the evaluation constitutes another influential factor. Verschekelen et al. [39] described a greater contribution of the superior costal level when respiratory maneuvers were carried out at the vital capacity level in a standing position compared to measurements at the vital capacity level in a supine position. These findings could also explain the variability in the results at the axillary level.

The inter-evaluator reproducibility in this project was similar to that of Malaguti et al. [9]. The ICCs were acceptable at the axillary level and good at the xiphoid and abdominal levels, with 95% IC. Muscular activation of the upper limbs may have contributed to lesser reproducibility at the axillary level. In healthy subjects, elevating the arm during everyday activities increases ventilatory activity, as the muscles implicated in the positioning of the arm decrease their participation in respiration, thus affecting the mechanics of the ventilatory effort [40]. These adjustments may apply to the present study, which could explain the acceptable reproducibility at the axillary level.

The intra- and inter-evaluator reproducibility was lower at the axillary level, which is considered below the clinically acceptable level. This level is based on the application of the measurements in clinical trials. In these studies the variability of group means is related to sample size. For that reason a 0.70 reliability threshold is appropriate [41].

On the other hand, the use of 0.7 threshold in the clinical scenario, would be limited due that the random variability of patient is greater that in clinical trial conditions. Additionally, intra- and inter-evaluator reproducibility was classified as clinically acceptable in xiphoid and abdominal levels. However, at the xiphoid level there was better inter-evaluator reproducibility than intra-evaluator, a finding that we consider a random effect because no clinical reasons are identified for such differences; and in any case, these differences do not represent changes in the decision to use or not the TI in the clinical or research context.

With regard to the external convergence construct validity, the analysis using the maximum value and the average of the two instances of the TI measurement and photogrammetry showed a correlation of between moderate and high at every level. The confidence intervals were wide at the three levels.

This positive correlation between the kinematic and TI analyses is based on the fact that both tests were evaluating the same construct: costal mobility, and the maneuvers used in the two cases are similar. Costal mobility results from the distensibility of tissue, and it could be inferred that both methods measure this biomechanical property at the three levels. Previous studies [9] have suggested that distensibility is greatest at the abdominal level, which could explain the high correlation observed in this study at that level.

The lesser correlation at the costal level (axillary and xiphoid) can be understood beginning with the modifications to the distribution of air in this area, responding to individual breathing patterns and the consequences of intermittent hyperinflation. In asthma, hyperinflation is considered intermittent, as it appears during crises and disappears during inter-crisis periods [38]. With regard to the type of pattern used, it was considered relevant to evaluate the correlation by allowing each subject to spontaneously maximize his/her respiration, which allowed for modified distributions of the volume of air in each test.

The consequences of hyperinflation with regard to the distribution of air in the rib cage have been studied by other authors [3], and it has been established that the volume of trapped air moves principally to the upper costal level, thus reducing distensibility [33]. This, together with the pathologic increased time taken to empty a lung of air and the local limitation to respiratory flow, produces hyperinflation in the other regions of the lung in patients with asthma [42].

The results of this study suggest that TI reliability and validity are similar when using measurements of the maximum and average values. The maximum value corresponds to a greater effort by a person to move lung volumes and capacities into and out of the rib cage, while the average value illustrates the typical form of costal mobility when the subject is requested to exert a greater-than-baseline respiratory effort [43].

For the above reasons, the TI can be used in clinical practice with people who can perform maximally at just one attempt, as well as those who require two or more attempts to obtain a result. In either case, a standardized protocol that includes every level of measurement should be considered, with an emphasis on explaining the maneuver and the commands or requests that are most effective at eliciting maximum force.

Some factors in the evaluation of the TI are outside the control of the evaluator, for example, sex, dynamic postural adjustments, breathing pattern, and muscular activation during the test [6], but they should be considered in the clinical setting when the effects of the disease or the results of the evaluation and the physiotherapeutic management are being analyzed. The evaluation of the effects of an intervention on costal mobility should include all these factors and should always be analyzed keeping in mind that statistically significant changes should be reflected in an improvement of the clinical condition.

Within the limits of the study, it was found that the TI’s psychometric properties were established for adults with stable asthma, for which reason the results are limited to people with these characteristics. It is recommended that the psychometric properties of these evaluations be assessed with other age groups, at different phases of the disease, and with other pathologies.

The TI measurements and measurements of costal kinematics were taken after requesting maximum respiratory force. However, lung volumes and capacities were not measured objectively using tools such as plethysmography. Thus, it was not possible to standardize the exact quantifiable volume when the measurements were taken. It is recommended that lung volumes and capacities be quantified using plethysmography to decrease variability in TI measurement in future research.

## Conclusions

Based on our review of the literature, this is the first study to evaluate the reliability and validity of perimetry in adults with stable asthma. Many works have demonstrated changes to respiratory mechanics in people with asthma, so this kind of evaluation should be routine for asthma patients.

The quality of the measurement techniques determines the quality of the research results and the decisions for the clinical management of these patients, and studies of reliability and validity help avoid errors in interpreting variables before and after interventions. In this study, the good reliability can be attributed to the standardization of the test and the use of a special session for familiarization. Nonetheless, it is important to remember that some aspects could not be controlled by the evaluator such as the subject’s sex and his/her dynamic postural adjustments.

A moderate to high correlation was found between the costal mobility variables, and the validity of the external convergent construct for the TI was established. This correlation stems from the fact that the same construct (costal mobility) was evaluated for both tests, and it depends on the distensibility of tissues. The elements that affect this correlation and that are not susceptible to control are individual breathing patterns and the consequences of the pathology for respiratory mechanics.

It can be concluded that the TI is a valid and reproducible measurement that can be used by health professionals during the physical examination of the thorax to evaluate thoracic mobility in adults with asthma, thus broadening their analysis of the respiratory mechanics in each case. This test can also be applied in controlled clinical trials to determine the effectiveness of therapeutic interventions for optimizing respiratory force in people suffering from this pathology.

## Abbreviations

BMI: Body mass index
CI95%: Confidence intervals at 95%
FEF: Forced expiratory flow
FEV_1_: Volume exhaled at the end of the first second
FVC: Forced vital capacity
ICC: Interclass correlation coefficient
PEF: Peak expiratory flow
TI: Thoracic index

## References

[1] Bateman ED, Hurd SS, Barnes PJ, Bousquet J, Drazen JM, FitzGerald M (2008). Global strategy for asthma management and prevention: GINA executive summary. Eur Respir J.

[2] Laghi F, Tobin MJ (2003). Disorders of the respiratory muscles. Am J Respir Crit Care Med.

[3] Gorini M, Iandelli I, Misuri G, Bertoli F, Filippelli M, Mancini M (1999). Chest wall hyperinflation during acute bronchoconstriction in asthma. Am J Respir Crit Care Med.

[4] Pedrolongo P, Gatti EM, Jamami M, Di Lorenzo BA, Costa D. Relação da medida da amplitude tóraco-abdominal de adolescentes asmáticos e saudáveis com seu desempenho físico. 2011;1:107–14. 24.

[5] Cihak-TR-2005-32.pdf [Internet]. [cited 2017 Oct 9]. Available from: http://cmp.felk.cvut.cz/ftp/articles/sara/Cihak-TR-2005-32.pdf.

[6] Romei M, Mauro AL, D’Angelo MG, Turconi AC, Bresolin N, Pedotti A (2010). Effects of gender and posture on thoraco-abdominal kinematics during quiet breathing in healthy adults. Respir Physiol Neurobiol.

[7] Davidson J, dos Santos AMN, Garcia KMB, Yi LC, João PC, Miyoshi MH (2012). Photogrammetry: an accurate and reliable tool to detect thoracic musculoskeletal abnormalities in preterm infants. Physiotherapy.

[8] Bockenhauer SE, Chen H, Julliard KN, Weedon J (2007). Measuring thoracic excursion: reliability of the cloth tape measure technique. J Am Osteopath Assoc.

[9] Malaguti C, Rondelli RR, de Souza LM, Domingues M, Dal Corso S (2009). Reliability of chest wall mobility and its correlation with pulmonary function in patients with chronic obstructive pulmonary disease. Respir Care.

[10] Caldeira V d S, Starling CCD, Britto RR, Martins JA, Sampaio RF, Parreira VF (2007). Precisão e acurácia da cirtometria em adultos saudáveis. J Bras Pneumol.

[11] Custers JWH, Arets HGM, Engelbert RHH, Kooijmans FTC, van der Ent CK, Helders PJM (2005). Thoracic excursion measurement in children with cystic fibrosis. J Cyst Fibros.

[12] Plaza Moral V (2015). GEMA4.0. Guía española para el manejo del asma. Arch Bronconeumol.

[13] Apter AJ (2012). Advances in adult asthma diagnosis and treatment and health outcomes, education, delivery, and quality in 2011: what goes around comes around. J Allergy Clin Immunol.

[14] Orozco LC (2010). Confiabilidad o de la consistencia, reproducibilidad, acuerdo y algo más. Medición en salud Diagnóstico y evaluación de resultados: un manual critico más allá de lo básico. primera edición.

[15] Puente Maestú L, García de Pedro J (2012). Lung function tests in clinical decision-making. Arch Bronconeumol.

[16] Troosters T, Casaburi R, Gosselink R, Decramer M (2005). Pulmonary rehabilitation in chronic obstructive pulmonary disease. Am J Respir Crit Care Med.

[17] Miller MR, Hankinson J, Brusasco V, Burgos F, Casaburi R, Coates A (2005). Standardisation of spirometry. Eur Respir J.

[18] Maestu L, Garcia J (2012). Lung function tests in clinical decision-making.

[19] Schlegelmilch RM, Kramme R (2011). Pulmonary Function Testing. Springer Handbook of Medical Technology [Internet].

[20] Pellegrino R, Viegi G, Brusasco V, Crapo RO, Burgos F, Casaburi R (2005). Interpretative strategies for lung function tests. Eur Respir J.

[21] Hernandez AG (2010). Tratado de nutricion / Nutrition Treatise: Composicion Y Calidad Nutritiva De Los Alimentos / composition and nutritional quality of foods. Ed Médica Panamericana.

[22] CDC. CDC Works 24/7 [Internet]. Centers for Disease Control and Prevention. 2017. Available from: https://www.cdc.gov/index.htm. [cited 2017 Jun 8].

[23] NHANES - National Health and Nutrition Examination Survey Homepage [Internet]. 2017. Available from: https://www.cdc.gov/nchs/nhanes/index.htm. [cited 2017 Oct 9].

[24] Heyward VH (2008). Evaluación de la aptitud física y prescripción del ejercicio. Ed Médica Panamericana.

[25] Pryor JA, Prasad AS. Physiotherapy for respiratory and cardiac problems: adults and paediatrics. London: Elsevier Health Sciences; 2008. p. 648.

[26] Pulsioximetría (medición de la saturación de oxígeno) [Internet]. [cited 2017 Oct 9]. Available from: https://www.elsevierclinicalskills.es/procedimientos/1129/pulsioximetr%C3%83%C2%ADa-medici%C3%83%C2%B3n-de-la-saturaci%C3%83%C2%B3n-de-ox%C3%83%C2%ADgen.

[27] Heyward VH. Evaluación y prescripción del ejercicio. Editorial Paidotribo; 2006. p. 284.

[28] [AQUI - epg4-76 ok.pdf [Internet]. [cited 2017 Oct 9]. Available from: http://biblioteca.univap.br/dados/INIC/cd/epg/epg4/epg4-76%20ok.pdf

[29] Linder W. Digital Photogrammetry [Internet]. Berlin, Heidelberg: Springer Berlin Heidelberg; 2009 [cited 2017 Oct 9]. Available from: http://link.springer.com/10.1007/978-3-540-92725-9

[30] Microsoft Word - pearson.doc - pearson2.pdf [Internet]. [cited 2017 Oct 9]. Available from: http://test.fisterra.com/gestor/upload/guias/pearson2.pdf

[31] L Orozco. Medición en salud. Diagnóstico y evaluación de resultados. Un manual crítico más allá de lo básico. 1 edición. Bucaramanga: Publicaciones UIS; 2010. p. 17–25.

[32] Lee L-J, Chang AT, Coppieters MW, Hodges PW (2010). Changes in sitting posture induce multiplanar changes in chest wall shape and motion with breathing. Respir Physiol Neurobiol.

[33] Courtney R (2009). The functions of breathing and its dysfunctions and their relationship to breathing therapy. Int J Osteopath Med.

[34] Lesmes JD (2007). Evaluación clínico-funcional del movimiento corporal humano. Ed Médica Panamericana..

[35] Kendall FP, McCreary EK, Provance PG. Muscles, testing and function: with posture and pain. USA: Williams & Wilkins; 1993. p. 451.

[36] Segizbaeva MO, Pogodin MA, Aleksandrova NP (2013). Effects of body positions on respiratory muscle activation during maximal inspiratory maneuvers. Adv Exp Med Biol.

[37] Burianová K, Vařeková R, Vařeka I (2008). The effect of 8 week pulmonary rehabilitation programme on chest mobility and maximal inspiratory and expiratory mouth pressure in patients with bronchial asthma. Acta Gymnica.

[38] Jardim JR, Mayer AF, Camelier A (2002). Músculos respiratorios y rehabilitación pulmonar en asmáticos. Arch Bronconeumol.

[39] Verschakelen JA, Demedts MG (1995). Normal thoracoabdominal motions. Influence of sex, age, posture, and breath size. Am J Respir Crit Care Med.

[40] Brown PI, Johnson MA, Sharpe GR (2014). Determinants of inspiratory muscle strength in healthy humans. Respir Physiol Neurobiol.

[41] Frost MH, Reeve BB, Liepa AM, Stauffer JW, Hays RD (2007). What Is Sufficient Evidence for the Reliability and Validity of Patient-Reported Outcome Measures?.

[42] Filippelli M, Duranti R, Gigliotti F, Bianchi R, Grazzini M, Stendardi L (2003). Overall contribution of chest wall hyperinflation to breathlessness in asthma. Chest.

[43] Corrigan C (2012). Mechanisms of asthma. Medicine (Baltimore).

