# Correction to: Thoracic index in adults with asthma: a study of validity and reliability

**DOI:** 10.1186/s12998-018-0201-3

**Published:** 2018-07-09

**Authors:** Yannely Serrano-villar, Eliana-isabel Rodríguez-grande

**Affiliations:** 1 Universidad Industrial de Santander Facultad de Salud, Bucaramanga, Santander Colombia; 2 Sede Quinta Mutis, Universidad del Rosario, Carrera 24N # 6, 3D-69 Bogotá, Colombia

## Correction

The original article [1] had 2 paragraphs which contained erroneous information. In this correction article the correct and incorrect information is shown.

- **Page 1**
  - **○ Incorrect:** The Thoracic Index (TI) is a useful tool for evaluating costal mobility as a component of respiritory mechanics in adults with asthma.
  - **○ Correct:** The Thoracic Index (TI) is a useful tool for evaluating costal mobility as a component of respiratory mechanics in adults with asthma.
- **Page 3**
  - **○ Incorrect**: Reflective markers were placed on the anatomical structures at each level, including the axillary level, the third anterior intercostal space in the front, and the fifth thoracic spinous process in the back; at the level of the xiphoid, the xiphoid process in the front and the tenth thoracic spinous process in the back; at the umbilical level, the umbilical scar in the front and the third thoracic spinous process in the back.
  - **○ Correct**: Reflective markers were placed on the anatomical structures at each level, including the axillary level, the third anterior intercostal space in the front, and the fifth thoracic spinous process in the back; at the level of the xiphoid, the xiphoid process in the front and the tenth thoracic spinous process in the back; at the umbilical level, the umbilical scar in the front and the third lumbar spinous process in the back.
